# The Connection between Czc and Cad Systems Involved in Cadmium Resistance in *Pseudomonas putida*

**DOI:** 10.3390/ijms22189697

**Published:** 2021-09-08

**Authors:** Huizhong Liu, Yu Zhang, Yingsi Wang, Xiaobao Xie, Qingshan Shi

**Affiliations:** Guangdong Provincial Key Laboratory of Microbial Culture Collection and Application, State Key Laboratory of Applied Microbiology Southern China, Institute of Microbiology, Guangdong Academy of Sciences, Guangzhou 510070, China; hzliucn@163.com (H.L.); zhangyu_1177@163.com (Y.Z.); wongvincy@163.com (Y.W.)

**Keywords:** *P. putida* KT2440, CzcRS two-component system, CadR regulator, gene regulation, cadmium resistance

## Abstract

Heavy metal pollution is widespread and persistent, and causes serious harm to the environment. *Pseudomonas putida*, a representative environmental microorganism, has strong resistance to heavy metals due to its multiple efflux systems. Although the functions of many efflux systems have been well-studied, the relationship between them remains unclear. Here, the relationship between the Czc and Cad systems that are predominantly responsible for cadmium efflux in *P. putida* KT2440 is identified. The results demonstrated that CzcR3, the response regulator of two-component system CzcRS3 in the Czc system, activates the expression of efflux pump genes *czcCBA1* and *czcCBA2* by directly binding to their promoters, thereby helping the strain resist cadmium stress. CzcR3 can also bind to its own promoter, but it has only a weak regulatory effect. The high-level expression of *czcRS3* needs to be induced by Cd^2+^, and this relies on the regulation of CadR, a key regulator in the Cad system, which showed affinity to *czcRS3* promoter. Our study indicates that the Cad system is involved in the regulation of the Czc system, and this relationship is important for maintaining the considerable resistance to cadmium in *P. putida*.

## 1. Introduction

Anthropogenic and geological activities release heavy metals into the environment, making heavy metal pollution a significant threat to human and ecosystem health [1,2]. With the rapid development of industries such as mining [3], plating [4], nano-metallic materials [5], and electronics [6], this problem is increasing. Common hazardous heavy metal pollutants include As, Cd, Co, Cr, Cu, Hg, Mn, Ni, Pb, etc. Some of these metal ions (e.g., Co^2+^, Cu^2+^, and Mn^2+^) are essential for biological processes at trace concentration [7]. To obtain these important metal ions, bacteria encode a variety of uptake transporters. These transporters are usually low-specific and driven by the chemiosmotic gradient across the cytoplasmic membrane [7]. For instance, the chemiosmotic transporter CorA mediates the influx of Mg^2+^, Co^2+^ and Ni^2+^ [8], and the Mn^2+^ and Zn^2+^ transporters allow Cd^2+^ to enter the cell [9,10]. This mode of transportation easily causes bacteria to uptake excessive metal ions and some toxic heavy metals in the environment contaminated by heavy metals. Excessive heavy metals in organisms can disturb metabolic functions through the denaturing of proteins, generating reactive oxygen species, and disrupting the intracellular ion balance [11,12,13].

It has been reported that the soil in some regions contains multiple heavy metals, such as Cd (0.1–3.6 mg/kg), Cr (51–207 mg/kg), Cu (14–109 mg/kg), and Pb (9.6–100 mg/kg) [14,15]. Bacteria in the environment are often exposed to heavy metals, thus they have evolved resistance systems to protect themselves from these hazardous substances. The sequestration of heavy metal ions by extracellular polymeric substances and metallothionein confers bacteria a certain degree of heavy metal tolerance [16,17]. Some bacteria can also transform heavy metal ions into non-toxic forms, for instance, Cd^2+^ can be immobilized by biosynthesizing CdS quantum dots in *Pseudomonas fragi* and Cd-containing nanoparticle inclusions in *Cupriavidus* species [18,19]. Alternatively, expulsion through efflux systems is a more common and effective way to protect bacteria from heavy metals. The transportation of different heavy metals is generally driven by one or several specific efflux pumps; therefore, bacteria usually encode many different types of efflux pump systems in the genome and plasmid [20,21]. The diversity of heavy metal resistant genes in a bacterial strain, which is partly promoted by horizontal gene transfer, results in multiple heavy metal resistant phenotypes [22].

The cadmium efflux system, which mainly consists of the Czc system and Cad system, is a well-characterized mechanism of heavy metal resistance in bacteria [23]. The Czc system is composed of CzcCBA, CzcRS, and CzcD. CzcCBA, a transporter across the inner and outer membranes, is assembled from the outer membrane protein CzcC, the membrane fusion protein CzcB, and the inner membrane protein CzcA [24]. It functions as a cation-proton antiporter to transport excess Cd^2+^, Zn^2+^, and Co^2+^ in the cytoplasm and periplasm to outside the cell [25]. The expression of *czcCBA* operon is regulated by the two-component system CzcRS. The regulatory effect of CzcRS can be activated by Cd^2+^, Zn^2+^, and Co^2+^ [26]. Structural and functional analysis showed that when the periplasmic sensor domain of the histidine kinase CzcS binds to Zn^2+^, the intracellular kinase domain of CzcS will autophosphorylate and then transmits the phosphate group to the intracellular response regulator CzcR [27]. Subsequently, the phosphorylated CzcR promotes the transcription of *czcCBA*. CzcD is a cation diffusion facilitator (CDF) protein family transporter located in the cytoplasmic membrane. It can also reduce Cd^2+^, Zn^2+^, and Co^2+^ accumulation in the cytoplasm, but only provides a small degree of resistance to metal ions compared to CzcCBA [28,29]. The Cad system contains the P-type ATPase superfamily efflux pump CadA, the ArsR/SmtB family regulator CadC, and the MerR family transcriptional regulator CadR. CadR has a high affinity to Cd^2+^ and a relatively weak affinity to Zn^2+^ and Pb^2+^ [30]. When the CadR dimer binds Cd^2+^, it can distort the targeted promoter, leading to transcription activation of the targeted genes [30]. CadA, which is expressed under the control of CadR, is an effective transporter for expelling Cd^2+^ in the cytoplasm to the periplasm by utilizing the energy from ATP decomposition [30,31]. CadC, which is encoded immediately downstream of *cadA* in *Staphylococcus aureus*, acts as a transcriptional repressor of *cadA-cadC* operon [32]. In some bacteria, such as *Ralstonia metallidurans* and *Pseudomonas putida*, the Czc and Cad systems are both important to confer full resistance to several heavy metals: the elimination of either system would significantly reduce resistance to Cd^2+^ and Zn^2+^ [33,34].

*P. putida* is a class of beneficial bacteria that exists widely in terrestrial and aquatic environments [35]. It can efficiently degrade various organic pollutants and promote plant growth [36,37]. Due to the development of genetic tools designed for genome editing, and the deep understanding of metabolic pathways, *P. putida* strains have been engineered as bacterial platforms for the biosynthesis of industrially relevant compounds such as cis,cis-muconic acid [38,39]. Some *P. putida* strains isolated from areas contaminated with heavy metal contaminants, such as *P. putida* CD2 and *P. putida* X4, show strong resistance to some hazardous heavy metals, especially cadmium [40,41]. These resistances are inseparable from the contribution of the Czc and Cad systems encoded in their genomes. However, so far, few studies have focused on the connection that evolved under heavy metal stress between these two types of efflux systems. In this study, the representative environmental model strain *P. putida* KT2440 [42] was used to explore the relationship between the Czc and Cad systems. The results provide insights into the regulatory connection between CadR, CzcRS3, and CzcCBA at a molecular level, and their roles in cadmium resistance of *P. putida*.

## 2. Results and Discussion

### 2.1. Involvement of Two-Component System CzcRS3 in Heavy Metal Resistance

**Figure 1:**
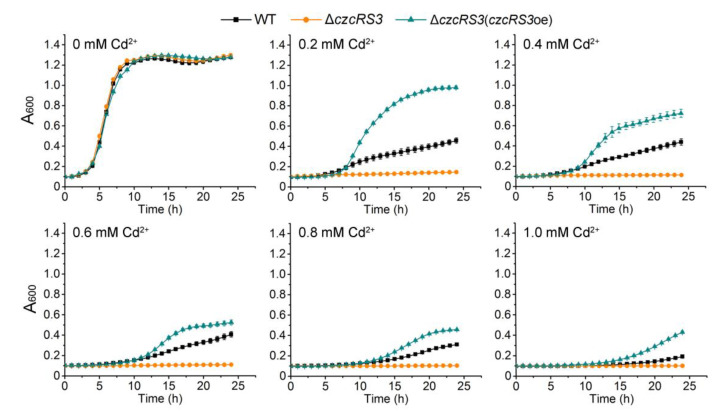
The role of *czcRS3* in *Pseudomonas putida* cadmium resistance. The wild-type *P. putida* KT2440 (WT, black), deletion Δ*czcRS3* (orange), and overexpressed Δ*czcRS3*(*czcRS*3oe) (blue, harboring pB403-*czcRS3*) cells were incubated in LB medium supplemented with CdCl_2_ at concentrations ranging from 0 to 1 mM. The absorbance at 600 nm (A_600_) was measured to evaluate the bacterial growth under cadmium stress. The data represent the mean ± standard deviation of three replicates.

Among the three *czcR* genes predicted in the *P. putida* KT2440 genome (*Pseudomonas* Genome Database version 20.2, https://www.pseudomonas.com, accessed on 3 September 2021), *czcR1* (*PP_0029*) and *czcR3* (*PP_1438*) are immediately followed by a cognate histidine kinase encoding gene (*czcS*), while *czcR2* (*PP_0047*) is orphaned [43]. In *P. putida* X4, the homologous operon of *czcRS1* could maintain a considerable expression level without metal ion inducers and is even repressed by Zn^2+^ and Cd^2+^, whereas the homologous operon of *czcRS3* needs to be induced by Cd^2+^ for expression [41]. This implies that *czcRS3* of *P. putida* KT2440 may be more related to cadmium resistance. Therefore, the role of this two-component system in heavy metal resistance, and the regulation of its expression, was the focus of this study. Firstly, the *czcRS3* was inactivated by homologous recombination, and the modifications in heavy metal resistance were tested. The MIC of Cd^2+^ in LB medium was reduced by four-fold in the *czcRS3* deletion mutant (Δ*czcRS3*), while no change was observed in the MICs of other heavy metals (Co^2+^, Cr^3+^, Cu^2+^, Mn^2+^, Ni^2+^, Pb^2+^, and Zn^2+^), compared to that in the wild-type (Table 1). When the cloned *czcRS3* was expressed under the control of the inducible *tac* promoter in Δ*czcRS3* (Δ*czcRS3*(*czcRS3*oe)), cadmium resistance was significantly increased, to a level that exceeded the wild-type level (Figure 1). These results indicate that CzcRS3 plays an important role in cadmium resistance of *P. putida* KT2440.

### 2.2. CzcRS3 Controls the Expression of Two CzcCBA Efflux Pumps

**Figure 2:**
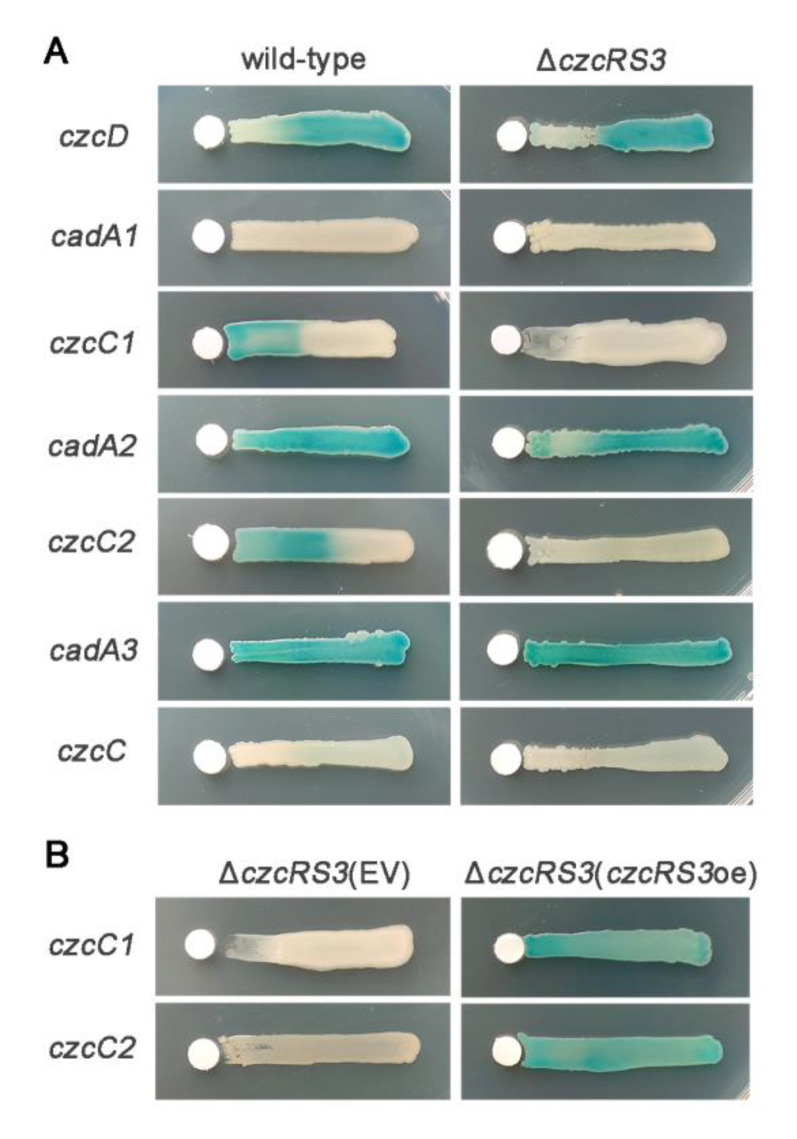
Role of CzcRS3 in the expression of efflux pump encoding genes. (**A**) The *lacZ* reporter plasmids carrying the promoter of indicated genes (the first gene of the operons) were introduced into wild-type KT2440 and deletion Δ*czcRS3*. (**B**) The empty vector pBBR1-403 (EV) and pB403-*czcRS3* (*czcRS3*oe) were introduced into the Δ*czcRS3* strains harboring the reporter plasmid for either *czcC1* or *czcC2*. The fresh bacterial cultures were streaked on the LB agar containing 40 μg/mL X-gal (5-bromo-4-chloro-3-indolyl β-D-galactopyranoside). The filter paper pieces on the left side were supplemented with 4 μL of 0.1 M CdCl_2_. The blue substance, which is the hydrolyzed product of X-gal by LacZ, indicates activation of the tested promoters. The photos were taken after 4 d of incubation.

The genome of KT2440 encodes several efflux pumps (*czcD*, *cadA1*, *cadA2*, *cadA3*, *czcCBA1*, *czcCBA2*, and *czcC-cusBA*) that have a clear or putative relationship with cadmium resistance [34,43,44]. To elucidate the decreased cadmium resistance of Δ*czcRS3*, the expression of these genes in Δ*czcRS3* was detected by promoter-fused *lacZ* (β-galactosidase encoding gene) reporter plasmids. As shown in Figure 2A, the expression of *cadA1* and *czcC-cusBA* was not detected in either wild-type or Δ*czcRS3*, regardless of exposure to Cd^2+^. Their expression might need to be induced by other factors. This was supported by the fact that *cadA1* maintained a very low or undetectable expression level without a divalent metal inducer, while Zn^2+^, but not Cd^2+^, was able to induce its expression [34]. In contrast, *czcD*, *cadA2*, and *cadA3* maintained significant expression levels in both the wild-type and Δ*czcRS3*, with or without Cd^2+^ (Figure 2A). There was no visible difference in the expression of these five genes between the wild-type and Δ*czcRS3*, suggesting that CzcRS3 did not regulate the expression of these efflux pumps.

The activation of *czcCBA1* and *czcCBA2* promoters in the wild-type could not be detected under the cadmium-free condition, but increased significantly upon exposure to Cd^2+^ (Figure 2A). However, when *czcRS3* was knocked out, these two operons were no longer induced by Cd^2+^, indicating that expression of *czcCBA1* and *czcCBA2* relied on CzcRS3 and Cd^2+^ (Figure 2A). To further verify this, *czcRS3* was supplemented to the mutant by a plasmid carrying the cloned *czcRS3* driven by the inducible *tac* promoter, which could avoid the interference from the regulators involved in *czcRS3* expression. As expected, the expression of *czcCBA1* and *czcCBA2* in Δ*czcRS3* was triggered by the expression of *czcRS3* (Figure 2B). Interestingly, the cells of Δ*czcRS3* with overexpressed *czcRS3* (Δ*czcRS3*(*czcRS3*oe)) on the cadmium-free side could also activate the expression of these two efflux pumps (Figure 2B). These results imply that CzcRS3 is the key regulator for *czcCBA1* and *czcCBA2* expression, and Cd^2+^ plays a role in other processes rather than the activation of their expression by CzcRS3. A previous study has investigated the roles of these two CzcCBA efflux pumps in heavy metal resistance, and showed that deletion of *czcA1* significantly reduced the resistance of the strain to Cd^2+^ and Zn^2+^ in minimal medium, whereas *czcA2* deletion did not affect the sensitivity to heavy metals [34]. This suggests that the decrease in cadmium resistance of Δ*czcRS3* is probably due to the blocking of *czcCBA1* expression.

### 2.3. CzcR3 Directly Binds to the Promoters of czcCBA1 and czcCBA2

**Figure 3:**
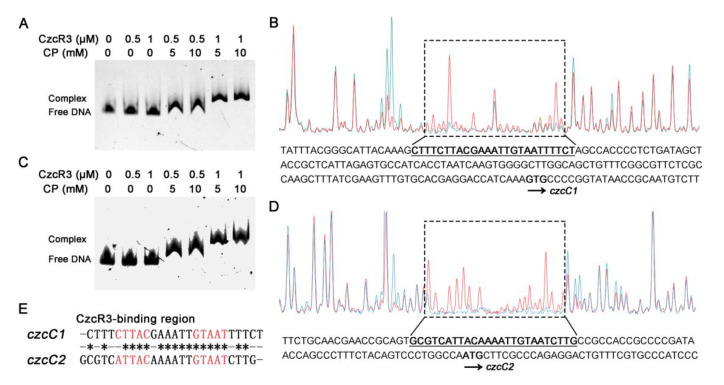
The binding of CzcR3 to the promoters of two *czcCBA* operons. (**A**,**C**) The FAM-tagged promoter probes of *czcC1* (**A**) and *czcC2* (**C**) were subjected to EMSA with the purified CzcR3 and carbamyl phosphate (CP). The hysteretic bands indicate the complex of CzcR3 and the probes. (**B**,**D**) The binding sites of CzcR3 on promoters of *czcC1* (**B**) and *czcC2* (**D**) were identified by Dnase I footprinting assay, in which the probe was incubated with CzcR3 (blue peak) or BSA (red peak, as control). The region in the dashed box, which displays the blue peak weaker than the red control, represents the binding region of CzcR3 on the promoter. The arrows indicate the translation start sites of *czcC1* (**B**) and *czcC2* (**D**). (**E**) The binding sequences of CzcR3 on *czcC1* and *czcC2* promoters show a high degree of similarity.

Response regulators function by binding to the targeted promoters and activating or inhibiting transcription events, after being phosphorylated by the cognate histidine kinase [45]. Although CzcR has a significant regulatory role in gene expression, its binding site on promoter has rarely been identified. Thus, the binding of CzcR3 to the promoter of *czcCBA1* and *czcCBA2* was explored. Electrophoresis analysis showed that purified CzcR3 was able to form a homodimer in vitro (Figure S1), which was expected since its cognate histidine kinase CzcS could also occur dimerization [27]. Since the response regulators displayed a much higher DNA-binding affinity after phosphorylation [26,46], carbamyl phosphate was used as a phosphate group donor, which could be used for in vitro phosphorylation of response regulators, such as ArcA [47] and NtrC [48]. The EMSA results showed that, in the presence of carbamyl phosphate, CzcR3 had an affinity for the promoters of *czcC1* and *czcC2* (Figure 3A,C). To further identify the binding site of CzcR3 on the promoters, a DNase I footprinting assay was performed. The results showed that, relative to the translation start site, CzcR3 bound to the regions from −120 to −144 bp for *czcC1* and −45 to −69 bp for *czcC2* (Figure 3B,D). There was a high degree of similarity between these two sequences, and they displayed a common inverted repeat sequence (aTTAC-N6-GTAAT) internally, in which the 6 bp spacer was likely to be rich in A/T (Figure 3E). This feature was also found in the DNA-binding sequence of CzcR in *Pseudomonas stutzeri* [49].

### 2.4. Identification of the Transcription Pattern of czcRS3

**Figure 4:**
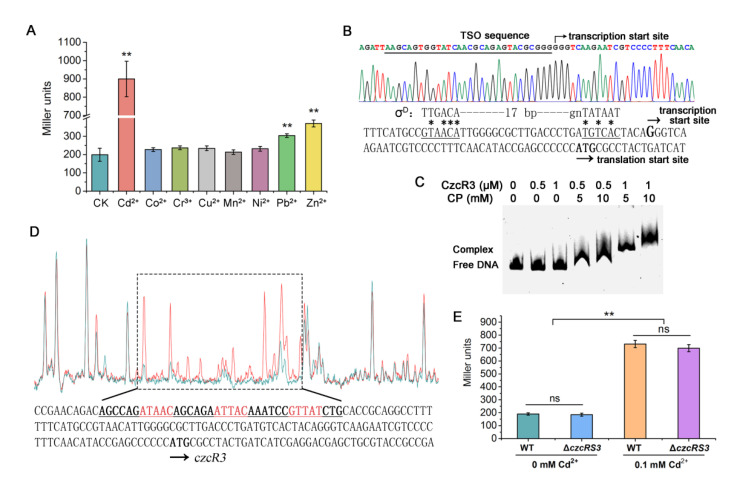
The regulation of *czcRS3* expression. (**A**) Induction of heavy metals on *czcRS3* expression. The expression level of *czcRS3* in wild-type KT2440 was detected by reporter plasmid pBRTZ-*czcR3* after treatment with heavy metals at a concentration of 1/5 MIC for 4 h. (**B**) Identification of the transcription start site of *czcRS3* operon by 5′-RACE assay. The underlined −10 and −35 elements show similarity to the recognition sequence of σ^D^ (* indicates the matched DNA bases). (**C**) The affinity of CzcR3 for *czcRS3* promoter in presence of carbamyl phosphate (CP). (**D**) Identification of the CzcR3-binding site on *czcRS3* promoter. The promoter probes were incubated with CzcR3 (blue peak) or BSA (red peak, as control) in DNase I footprinting assay. The region in the dashed box represents the binding region of CzcR3 on the promoter. (**E**) The regulatory role of CzcRS3 in self-expression. The data represent the mean ± standard deviation of three replicates. The significant difference was determined by Student’s *t*-test (** *p* < 0.01; ns, non-significant, *p* > 0.05).

To determine the role of CzcRS3 in cadmium resistance, the regulation of the *czcRS3* operon was studied. The expression level of *czcRS3* in the wild-type strain was evaluated by the reporter plasmid for *czcR3*. When heavy metals were absent, the *czcRS3* promoter exhibited low transcription activity (Figure 4A). Several heavy metals, including Cd^2+^, Co^2+^, Cr^3+^, Cu^2+^, Mn^2+^, Ni^2+^, Pb^2+^, and Zn^2+^, were used as inducers, at a concentration of 1/5 MIC. Among these inducers, only Cd^2+^ could strongly induce *czcRS3* expression, reflecting the important relationship between CzcRS3 and cadmium resistance. Pb^2+^ and Zn^2+^ also displayed an inducing effect on *czcRS3* expression, but much weaker than Cd^2+^ (Figure 4A).

Subsequently, the transcription start site of *czcRS3* was identified by a 5′-RACE assay, which showed that it was located 39 bp upstream of the translation start site of *czcR3* (Figure 4B). The −10 and −35 elements of this transcription start site displayed similarity to the recognition sequence of sigma factor σ^D^ [50]. However, there were several mismatched nucleotides in the critical −10 element, suggesting that efficient transcription of *czcRS3* might require the assistance of a positive regulator [51].

Analysis of the *czcRS3* promoter indicated a potential CzcR3 binding site in the −116 to −101 bp region, relative to the first codon of *czcR3*, which showed a high similarity to the CzcR3-binding sites on the *czcC1* and *czcC2* promoters. EMSA verified the binding ability of CzcR3 to its own promoter (Figure 4C), while DNase I footprinting assay showed that CzcR3 protected the region between −98 and −133 bp, relative to the first codon of *czcR3* (Figure 4D). It should be noticed that this 36 bp protected region was much longer than the CzcR3-protected region on *czcC1* and *czcC2* promoters, which were both approximately 25 bp (Figure 3B,D). Interestingly, this long protected region contained three putative CzcR3 recognition motifs (ATAAC, ATTAC, and GTTAT). Whether the binding complex contained two or three CzcR3 monomers could not be determined, since the structure of CzcR3 or its interaction with DNA has not yet been elucidated. To test the regulatory role of CzcRS3 on its encoding operon, the reporter plasmid pBRTZ-*czcR3* was introduced into Δ*czcRS3*. Surprisingly, the activity of *czcR3* promoter in Δ*czcRS3* was not significantly different from that in the wild-type (Figure 4E). Under induction by Cd^2+^, both the wild-type and Δ*czcRS3* could effectively activate the *czcR3* promoter (Figure 4E), suggesting that the unconventional binding of CzcR3 to its promoter had no obvious regulatory effect. This also implies that there are other regulators involved in *czcRS3* expression.

### 2.5. Cd^2+^ Induces czcRS3 Expression through CadR

**Figure 5:**
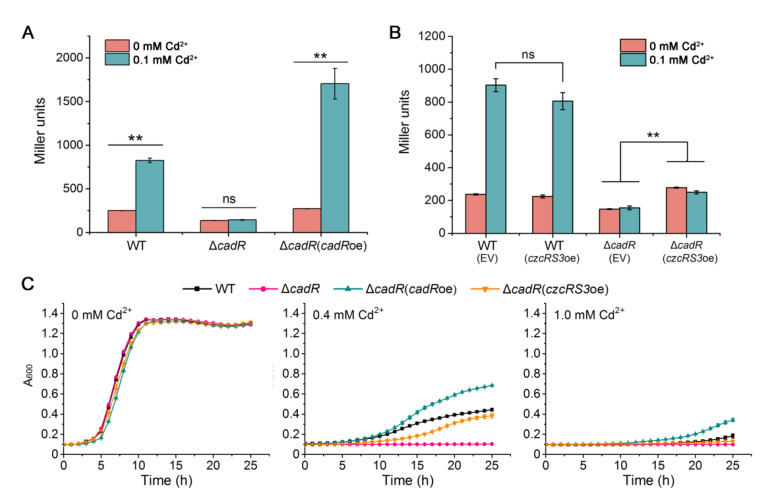
Involvement of CadR in *czcRS3* expression. (**A**) The expression level of *czcRS3* in wild-type KT2440 (WT), deletion Δ*cadR*, and overexpressed Δ*cadR*(*cadR*oe) which harbors pB403-*cadR*. (**B**) The expression level of *czcRS3* in KT2440 strains with overexpressed *czcRS3*. The activity of β-galactosidase expressed from pBRTZ-*czcR3* was measured after treatment with or without CdCl_2_ for 4 h. (**C**) The growth of Δ*cadR* with overexpressed *cadR* (*cadR*oe) or *czcRS3* (*czcRS3*oe) in LB medium supplemented with CdCl_2_ at the indicated concentrations. The data represent the mean ± standard deviation of three replicates. The significant difference was determined by Student’s *t*-test (** *p* < 0.01; ns, non-significant, *p* > 0.05).

CadR is a well-characterized transcriptional regulator that responds to Cd^2+^, Zn^2+^, and Pb^2+^ in *P. putida* KT2440 [30,52]. Along with the fact that Cd^2+^, Zn^2+^, and Pb^2+^ could induce *czcRS3* expression (Figure 4A), it is possible that CadR participates in *czcRS3* regulation. To confirm this hypothesis, the *cadR* deletion mutant (Δ*cadR*) was constructed, and the expression of *czcRS3* was determined in this mutant. As expected, the promoter of *czcRS3*, regardless of the presence of Cd^2+^, could not be activated in Δ*cadR* (Figure 5A). When Δ*cadR* was complemented with *cadR* (Δ*cadR*(*cadR*oe)), the activity of *czcRS3* promoter was restored to the wild-type level, and increased significantly upon exposure of 0.1 mM Cd^2+^ (Figure 5A). This indicates that CadR is the key regulator in response to Cd^2+^ to activate the expression of *czcRS3*.

To test whether the binding of CzcR3 on its own promoter had physiological significance in the context of knocking out *cadR*, the plasmid carrying the inducible *czcRS3* was introduced into Δ*cadR*, as well as the wild-type. Overexpression of *czcRS3* slightly activated the *czcR3* promoter in Δ*cadR*, and this activation was not dependent on supplementation with Cd^2+^ (Figure 5B). In comparison, it could not cause a significant change in the expression level of *czcRS3* in the wild-type (Figure 5B). The weak activation effect of the overexpressed CzcRS3 in *czcRS3* expression is possibly masked by CadR in the wild-type. The effect of overexpressed CzcRS3 on cadmium resistance was also tested under the background of Δ*cadR*. The cadmium resistance was significantly reduced after *cadR* was knocked out (Figure 5C), because *cadR* knockout would block the activation of *cadA*, which encodes a major cadmium efflux pump [34,52]. Complementation with *cadR* increased the cadmium resistance of Δ*cadR* to the wild-type level, while overexpression of *czcRS3* could also restore the cadmium resistance of Δ*cadR* to a certain extent (Figure 5C). This indicates that the regulatory effect of CadR on cadmium resistance partly depends on CzcRS3. In other words, CzcRS3 is a downstream regulator of CadR, and together they endow *P. putida* KT2440 with strong resistance to cadmium.

### 2.6. CadR Binds to czcRS3 Promoter Directly

**Figure 6:**
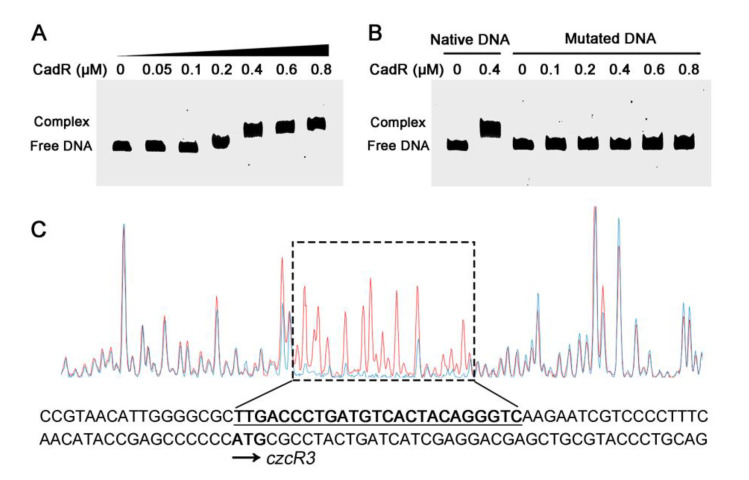
CadR directly binds to the *czcRS3* promoter. The native (**A**) and mutated (**B**) probes of *czcRS3* promoter were subjected to EMSA with the purified CadR. (**C**) The binding site of CadR on *czcRS3* promoter was identified by DNase I footprinting assay, in which the native probe was incubated with CadR (blue peak) or BSA (red peak, as control). The region in the dashed box represents the binding region of CadR on *czcRS3* promoter.

To investigate whether CadR regulated *czcRS3* expression directly, the His-tagged CadR was purified and its affinity for *czcRS3* promoter was tested. The EMSA result showed that the purified CadR was able to bind to the DNA fragment containing the *czcRS3* promoter (Figure 6A). This binding process did not require the participation of Cd^2+^, in agreement with previous work [30]. Furthermore, the DNase I footprinting assay identified the region protected by CadR from DNase I, which was located at −35 to −59 bp, relative to the first codon of *czcR3* (Figure 6C). An obvious inverted repeat sequence (CTTGACCCTG-N9-CAGGGTCAAG) was found over this region. The sequence of CadR-binding region on *czcRS3* promoter is highly similar to that on the *cadA* promoter [30], further supporting this result. To verify the CadR-binding region on *czcRS3* promoter, several base mutations were introduced in the promoter fragment (TTGACAAAG-N9-CAAAGTC) for EMSA. As expected, the point-mutated fragment could not interact with CadR (Figure 6B). These results indicate that CadR regulates *czcRS3* by directly binding its promoter.

Since CadR functions in a homodimer form, its binding site on DNA shows the characteristic of an inverted repeat. The binding of CadR to the promoter does not require metal ions; however, the CadR structure would change after binding to Cd^2+^, resulting in a distortion of the DNA and a reduced distance between the −10 and −35 elements of the promoter. This further leads the promoter to change from a repressed state to an activated state [30]. Although CadR can interact with Cd^2+^, Pb^2+^, and Zn^2+^, its binding affinity for Pb^2+^ and Zn^2+^ is much weaker than for Cd^2+^ [30]. Therefore, Cd^2+^ can strongly induce expression of the CadR-regulated genes, whereas Pb^2+^ and Zn^2+^ have weaker effects [52]. This also explains the differences in degrees of induction in *czcRS3* expression by Cd^2+^, Pb^2+^, and Zn^2+^ (Figure 4A).

## 3. Materials and Methods

### 3.1. Bacterial Strains and Growth Conditions

All bacterial strains and plasmids used in this study are listed in Table S1. Throughout the study, the *P. putida* strains were grown in Lysogeny broth (LB) medium at 28 °C, and the *E. coli* strains were grown in LB medium at 37 °C. When required, antibiotics (purchased from Sangon Biotech, Shanghai, China) were used at the following concentrations: chloramphenicol (25 mg/L), gentamycin (20 mg/L), tetracycline (20 mg/L), kanamycin (50 mg/L) and ampicillin (150 mg/L). Analytical-grade salts of CdCl_2_·2.5H_2_O, CoCl_2_·6H_2_O, CrCl_3_·6H_2_O, CuCl_2_·2H_2_O, MnCl_2_·4H_2_O, NiCl_2_·6H_2_O, Pb(NO_3_)_2_, and ZnCl_2_ (purchased from Macklin, Shanghai, China) were used to prepare 1 M stock solutions, which were sterilized by filtration.

### 3.2. Construction of Strains and Plasmids

Primers for mutant construction are listed in Table S2. To construct the knockout plasmids, the upstream and downstream regions of *czcRS3* and *cadR*, which were amplified from KT2440 genome by polymerase chain reaction (PCR), were fused into SacI-digested pDS3.0 [53] using the ClonExpress II one step cloning kit (Vazyme, Nanjing, China), yielding pDS-*czcRS3* and pDS-*cadR*, respectively. The plasmid in *E. coli* S17-1 was transferred to wild-type *P. putida* KT2440 for allelic exchange, and *sacB* counter-selection was used to select the mutants as described in detail previously [53,54]. Finally, Δ*czcRS3* and Δ*cadR* were obtained.

To construct the reporter plasmids, the 300–600 bp DNA fragments containing the promoter region of the tested genes were amplified using the primers for promoter in Table S2, and the fragments were ligated to XbaI- and PstI-digested pBRTZ, which contains the promoter-less β-galactosidase encoding gene *lacZ* [55], yielding the reporter plasmids for *czcD*, *cadA1*, *czcC1*, *cadA2*, *czcR3*, *czcC2*, *cadA3*, and *czcC* (Table S1), respectively. To construct the overexpression plasmids, the DNA fragments containing *czcRS3* or *cadR* encoding sequence were amplified using the primers for gene cloning in Table S2, and the fragments were ligated to EcoRI- and BamHI-digested pBBR1-403 [56] to yield pB403-*czcRS3* and pB403-*cadR*, respectively. When needed, isopropyl β-D-1-thiogalactopyranoside (IPTG) was used as the inducer of *tac* promoter at a concentration of 0.1 mM. The plasmids above were hosted in *E. coli* S17-1, and they were transferred to derivative strains of *P. putida* KT2440 by biparental mating. To construct the plasmids for protein purification, the encoding sequence of *czcR3* and *cadR* were amplified using the primers for protein purification in Table S2, and the fragments were ligated to NcoI- and XhoI-digested pET28a to yield pET28a-*czcR3* and pET28a-*cadR*, respectively. These plasmids were hosted in *E. coli* BL21(DE3).

### 3.3. Test for Susceptibility to Heavy Metals

The overnight cultures of *P. putida* strains were diluted with fresh LB medium to an OD_600_ of 0.005, and the metal salts were also serially diluted by 2-fold in LB medium. The diluted culture (75 μL) was mixed with an equal volume of diluted metal salts or antibiotics in a 96-well plate. The mixture was incubated at 28 °C for 24 h. The minimum inhibitory concentration (MIC) was defined as the lowest concentration of antibiotic with no visible bacterial growth [57]. In the growth assays, the diluted cultures containing Cd^2+^ were incubated in Spark 20M microplate reader (Tecan, Männedorf, Switzerland) and the absorbance at 600 nm (A_600_) was measured at intervals of 1 h.

### 3.4. Measurement of β-Galactosidase Activity

An overnight culture of KT2440 strains harboring the reporter plasmid was inoculated in fresh LB medium (1:100). To reduce the effect of the hazardous heavy metals on growth, the cultures were pre-incubated for 8 h, and then treated with heavy metal ions for 4 h. The β-galactosidase activity was measured according to the procedures described previously [54,58]. Briefly, the reaction system contained 50 μL culture samples, 450 μL Z buffer (60 mM Na_2_HPO_4_, 40 mM NaH_2_PO_4_, 10 mM KCl, 1 mM MgSO_4_ and 50 mM β-mercaptoethanol), 25 μL of 1 mg/mL sodium dodecyl sulfate (SDS), 50 μL chloroform, and 100 μL of 4 mg/mL 2-nitrophenyl-β-D-galactopyranoside (ONPG). The reaction proceeded at 28 °C and was terminated by 250 μL of 1 M Na_2_CO_3_. After centrifugation, Absorbance at 420 nm (A_420_) of the supernatant was measured, and A_600_ of the bacterial culture before lysis was also measured. The β-galactosidase activity was calculated as: Miller units = 1000 × (A_420_/A_600_/volume/time).

### 3.5. Identification of Transcription Start Site

Total RNA was isolated from wild-type KT2440 culture after treatment with 0.2 mM Cd^2+^ for 4 h. The 5′-rapid amplification of cDNA ends (5′-RACE) was performed using the vaccinia capping enzyme (VCE, NewEnglandBiolabs, Ipswich, MA, USA) to add a 5′ end cap structure to the RNA sample [59]. The RNA sample was then treated by DNase I (Takara, Japan) to remove the residual genome DNA and purified by standard ethanol precipitation as described in the manual for DNase I. First-strand cDNA was synthesized using HiScript II reverse transcriptase (Vazyme, China) and primer *czcRS3*-RC (Table S2). Then, the template-switching oligonucleotide TSO-RNA (Table S2) was used as the template for reverse transcriptase to add the adapter sequence to the 3′ end of cDNA. The final cDNA was amplified using primers *czcRS3*-RC and TSO-DNA (Table S2), and the product was linked to pMD19-T (Takara, Japan). The transcription start site of *czcRS3* operon was identified through sequencing.

### 3.6. Purification of His-Tagged CzcR3 and CadR

The *E. coli* BL21(DE3) strains harboring pET28a-*czcR3* or pET28a-*cadR* were incubated in 500 mL LB medium with shaking at 220 rpm to an OD_600_ of 0.5. The cultures were treated with 0.5 mM IPTG at 20 °C for 6 h. The cells were collected by centrifugation and resuspended in 20 mL of lysis buffer (10 mM Tris-HCl at pH 8.0, 50 mM NaCl, 10% *v*/*v* glycerol). After the cells were lysed by a high-pressure homogenizer, the His-tagged CzcR3 and CadR were purified using Ni-NTA spin columns (BBI Life Sciences, China), according to the directions from the manufacturer. The His-tagged proteins were eluted with E250 buffer (10 mM Tris-HCl at pH 8.0, 250 mM NaCl, 10% *v*/*v* glycerol, 250 mM imidazole). The elution samples were dialyzed to remove imidazole.

### 3.7. Generation of Fluorescent Probes of Promoters

The primers for the generation of the FAM-tagged probes are listed in Table S2. Primers M13F-*czcR3*pS and *czcR3*pA2 were used to amplify the fragment containing the *czcR3* promoter in the first round of PCR, and then primers M13F-fam and *czcR3*pA2 were used to add the fluorescent 6-carboxyfluorescein phosphoramidite (6-FAM) tag to the fragment in the second round of PCR. After the purification, the native probe for EMSA and DNase I footprinting assays was obtained. The FAM-tagged probes for *czcC1* and *czcC2* were generated following the same steps but using their specific primers. To generate the mutated probe of *czcR3* promoter, two fragments, which were amplified from genome DNA by M13F-*czcR3*pS/*czcR3*p-mA and *czcR3*pA2/*czcR3*p-mS, respectively, were spliced by overlap extension PCR using M13F-fam and *czcR3*pA2.

### 3.8. Electrophoretic Mobility Shift Assay (EMSA)

In EMSA, 10 nM of FAM-tagged probe was mixed with increasing concentrations of the purified protein (CzcR3 and CadR) in a 20 μL binding buffer system (10 mM Tris-HCl at pH 8.0, 50 mM KCl, 5 mM MgCl_2_, 5% *v*/*v* glycerol). In the binding system for CzcR3, the carbamyl phosphate was used as the donor of phosphate group. After 20 min of incubation at 28 °C, 15 μL of the sample was loaded onto a 5% (*w*/*v*) polyacrylamide gel and electrophoresed in TG buffer (12.5 mM Tris-HCl at pH 8.3, 96 mM glycine) at 100 V on ice for 90 min. The gels were photographed with ChemiDocXRS + (BioRad, Hercules, CA, USA).

### 3.9. DNase I Footprinting Assay

In DNase I footprinting assay, 40 nM of the FAM-tagged probe was mixed with about 2 μM of the purified protein or bovine serum albumin (BSA, negative control) in a 200 μL binding buffer system. After 20 min of incubation at 28 °C, the samples were treated with 0.3 U DNase I at room temperature for 5 min, and the reaction was quenched by treatment with 100 μL phenol-chloroform (1:1, *v*/*v*) and 80 °C heating. After centrifugation, the supernatant was mixed with 500 μL ethanol and 20 μL of 3 M sodium acetate (pH 5.2). The DNA was precipitated by centrifugation and dissolved in 20 μL ddH_2_O. The DNA samples were analyzed by 3730XL DNA Sequencer and the data was processed by Peak Scanner Software v1.0 (Applied Biosystems, Waltham, MA, USA).

## 4. Conclusions

**Figure 7:**
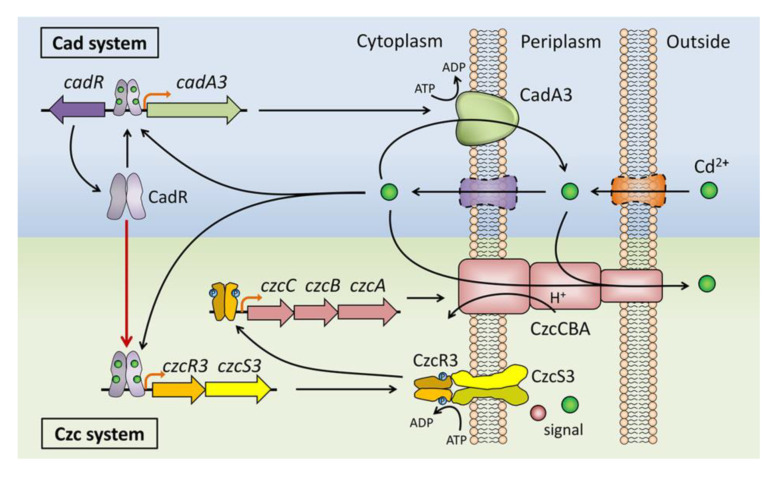
Cooperation between Czc and Cad systems confers *P. putida* the resistance to cadmium.

In this study, the role of a two-component system CzcRS3 in cadmium resistance of *P. putida* KT2440 was investigated and confirmed that CzcRS3 regulated the expression of two CzcCBA efflux pump operons by directly binding to their promoters. The regulation of *czcRS3* expression was also explored and determined that CzcR3 bound to its own promoter in an atypical way, but this did not have a significant effect on transcription. Importantly, the expression of *czcRS3* was directly regulated by CadR in response to Cd^2+^, Zn^2+^, and Pb^2+^, revealing the regulatory relationship between the Cad and Czc systems. Along with previous studies, these results support that these two systems essentially belong to a biological pathway (Figure 7). In this pathway, Cd^2+^ enters the cell and combines with CadR, and thereby CadR can promote the expression of the CadA3 efflux pump [30] and CzcRS3. CadA3 functions in the transport of Cd^2+^ from the cytoplasm to the periplasm [34], and CzcRS3 is responsible for activating the expression of two CzcCBA efflux pumps. CzcCBA can expel, not only part of the intracellular Cd^2+^, but also the Cd^2+^ in the periplasm that includes the Cd^2+^ exported from the cytoplasm by CadA, to the outside [33]. These two efflux systems, including their regulators, form effective cooperation to protect the *P. putida* from toxic cadmium in the environment.

## Figures and Tables

**Table 1:** Minimum inhibitory concentration (MIC) of heavy metals for wild-type *P. putida* KT2440 and Δ*czcRS3* in LB medium.

Metal Ion (mM)	Cd^2+^	Co^2+^	Cr^3+^	Cu^2+^	Mn^2+^	Ni^2+^	Pb^2+^	Zn^2+^
wild-type	1	1	4	8	16	8	8	8
Δ*czcRS3*	0.25	1	4	8	16	8	8	8

## Data Availability

The data presented in this study are available on request from the corresponding author.

## Supplementary Materials

The following are available online at https://www.mdpi.com/article/10.3390/ijms22189697/s1.
