# Supplementary material for: The Connection between Czc and Cad Systems Involved in Cadmium Resistance in Pseudomonas putida
Source: Int J Mol Sci. 2021 Sep 8;22(18):9697. doi: 10.3390/ijms22189697 (PMC8469834; doi:10.3390/ijms22189697)
Supplement: Supplementary file 1 [file ijms-22-09697-s001.zip › ijms-1363225-supplementary.pdf]

Supplementary Materials

The connection between Czc and Cad systems involved in cadmium resistance in *Pseudomonas putida*

Huizhong Liu, Yu Zhang, Yingsi Wang, Xiaobao Xie *, Qingshan Shi *

Guangdong Provincial Key Laboratory of Microbial Culture Collection and Application, State Key Laboratory of Applied Microbiology Southern China, Institute of Microbiology, Guangdong Academy of Sciences, Guangzhou 510070, China; hzliucn@163.com (H.L.); zhangyu_1177@163.com (Y.Z.); wongvincy@163.com (Y.W.)

* Correspondence: xiexb@gdim.cn (X.X.); jigan@gdim.cn (Q.S.)

Table S1. The strains and plasmids used in this study.

Strains and plasmids	Description	Reference
Strains		
E. coli S17-1	*RP4*$^{-}$, *mob*$^{+}$, *λpir*, host for plasmid construction	Lab stock
E. coli BL21(DE3)	*F*$^{-}$, *ompT, hsdS* (*rB*$^{-}$ *mB*$^{-}$), *gal, dcm* (DE3), host for protein expression	Vazyme Biotech
P. putida KT2440	Wild-type *Pseudomonas putida* KT2440	[1]
P. putida Δ*czcRS3*	*czcRS3* (*PP_1437-1438*) deletion mutant of KT2440	This study
P. putida Δ*cadR*	*cadR* (*PP_5140*) deletion mutant of KT2440	This study
Plasmids		
pDS3.0	Suicide vector, Gm^{R}, *R6K ori*, *sacB*	[2]
pDS-*czcRS3*	Knockout vector for *czcRS3*	This study
pDS-*cadR*	Knockout vector for *cadR*	This study
pBBR1-403	Expression vector, Gm^{R}, *lacI*, *tac* promoter	[3]
pB403-*czcRS3*	pBBR1-403 carrying complete *czcRS3*	This study
pB403-*cadR*	pBBR1-403 carrying *cadR*	This study
pBRTZ	Reporter vector, Tet^{R}, promoter-less *lacZ*	[4]
pBRTZ-*czcD*	pBRTZ carrying the promoter of *czcD* (*PP_0026*)	This study
pBRTZ-*cadA1*	pBRTZ carrying the promoter of *cadA1* (*PP_0041*)	This study
pBRTZ-*czcC1*	pBRTZ carrying the promoter of *czcC1* (*PP_0045*)	This study
pBRTZ-*cadA2*	pBRTZ carrying the promoter of *cadA2* (*PP_0586*)	This study
pBRTZ-*czcR3*	pBRTZ carrying the promoter of *czcR3* (*PP_1438*)	This study
pBRTZ-*czcC2*	pBRTZ carrying the promoter of *czcC2* (*PP_2408*)	This study
pBRTZ-*cadA3*	pBRTZ carrying the promoter of *cadA3* (*PP_5139*)	This study
pBRTZ-*czcC*	pBRTZ carrying the promoter of *czcC* (*PP_5385*)	This study
pET28a	Expression vector, Kan^{R}, *lacI*, *T7* promoter	Lab stock
pET28a-*czcR3*	pBRTZ carrying the encoding sequence of *czcR3*	This study
pET28a-*cadR*	pBRTZ carrying the encoding sequence of *cadR*	This study

Table S2. The primers used in this study.

Primers	Sequence (5′ to 3′)
Primers for mutant construction	
*czcRS3*upS	AGGTACCGCATGCGATATCGAGCTCATGCAGTCGCTGCTGGTCTT
*czcRS3*upA	CCAGGTCATACGCCTGTTGG
*czcRS3*dnS	CCAACAGGCGTATGACCTGGGCGCTTGTTCGAGCGGTTTT
*czcRS3*dnA	TTTGTGGAATTCCCGGGAGAGCTCAAGGTGGCCTGGATGTGGCA
*cadR*upS	AGGTACCGCATGCGATATCGAGCTCACGCCAAGCACGCGGTTGAT
*cadR*upA	CATGGGAATGTTCGGTATCC GCTCGTAGTAGCGGATGGTTTC
*cadR*dnS	CGGATACCGAACATTCCCATG
*cadR*dnA	TTTGTGGAATTCCCGGGAGAGCTCGCACGCCGTTCAACCTCACC
Primers for promoter	
*czcD*pS	CACCGCGGTGGCGGCCGCTCTAGAGAGCAATATGATCGCCATGG
*czcD*pA	TGAATGAGATTTAGTCATCTGCAGGCTGCCATGGTCATGATTTG
*cadA1*pS	CACCGCGGTGGCGGCCGCTCTAGATCGTACGTCAGTCGCAGGAC
*cadA1*pA	TGAATGAGATTTAGTCATCTGCAGAGGGCGTTCAAGCAAACTCT
*czcC1*pS	CACCGCGGTGGCGGCCGCTCTAGA CCGCCAAATACAGTGGTACTT
*czcC1*pA	TGAATGAGATTTAGTCATCTGCAGGACATTGCGGTTATACCGGGG
*cadA2*pS	CACCGCGGTGGCGGCCGCTCTAGACCTCCTTCAGGTTTTTCACAA
*cadA2*pA	TGAATGAGATTTAGTCATCTGCAGCAGGTCGTATGTGGTGGATGC
*czcR3*pS	CACCGCGGTGGCGGCCGCTCTAGACCACCTCACGCGCCTTCTC
*czcR3*pA	TGAATGAGATTTAGTCATCTGCAGGGTACGCAGCTCGTCCTCG
*czcC2*pS	CACCGCGGTGGCGGCCGCTCTAGAGAGGGTTTCGTGGCCGTACT
*czcC2*pA	TGAATGAGATTTAGTCATCTGCAGGAAACAGTCCTCTGGGCGAA
*cadA3*pS	CACCGCGGTGGCGGCCGCTCTAGAAAGGTCAGCCGCTCCACAT
*cadA3*pA	TGAATGAGATTTAGTCATCTGCAGGTGTTCGTGGCTGACAGGC
*czcC*pS	CACCGCGGTGGCGGCCGCTCTAGATCGCTTTGGCCTACACTCG
*czcC*pA	TGAATGAGATTTAGTCATCTGCAGCCCTGAATGGGAGCATAGG
Primers for gene cloning	
*czcRS3*oeS	ATTTCACACAGGAAACAGAATTCATGCGCCTACTGATCATCGAG
*czcRS3*oeA	GATCCGCCAAAACAGCCAAGCTTTTATGCCGATGCCGAAACC
*cadR*oeS	ATTTCACACAGGAAACAGAATTCATGAAGATCGGAGAACTGGCC
*cadR*oeA	GATCCGCCAAAACAGCCAAGCTT CCGATGGCGGTCTGATAGATC
Primers for protein purification	
*cadR*s	TTTAAGAAGGAGATATACCATGAAGATCGGAGAACTGGCC
*cadR*a	GTGGTGGTGGTGGTGCTCGAGATGCCCGTGACTCCGCCCC
*czcR3*s	ACTTTAAGAAGGAGATATACCATGCGCCTACTGATCATCGAG
*czcR3*a	TGGTGGTGGTGGTGGTGCTCGAGAAGGCGAGCCTCAAGTACGTA
Primers for FAM-tagged probe	
M13F-fam	TGTAAAACGACGGCCAGT (5′ end contains 6-FAM tag)
M13F-*czcC1*pS	TGTAAAACGACGGCCAGT GTATCGAAATCATCGTGTC
*czcC1*pA2	TGCGGTTATACCGGGGCAC
M13F-*czcC2*pS	TGTAAAACGACGGCCAGT GATTATCGAACTTATTGGGC
*czcC2*pA2	AGCATTGGCCAGGGACTGTA
M13F-*czcR3*pS	TGTAAAACGACGGCCAGTTACAGGCGCGGGTTTACTCG
*czcR3*pA2	CATGGGGGGCTCGGTATGT
*czcR3*p-mS	TTGGGGCGCTTGACAAAGATGTCACTACAAAGTCAAGAATCGTC
*czcR3*p-mA	GACGATTCTTGACTTTGTAGTGACATCTTTGTCAAGCGCCCCAA
Primers for 5′-RACE	
czcRS3-RC	TCGACCACGTAACCGTTCTCGCGAA
TSO-DNA	AAGCAGTGGTATCAACGCAGAGTACGCGGG
TSO-RNA	AAGCAGUGGUAUCAACGCAGAGUACGCGGG (oligo RNA)

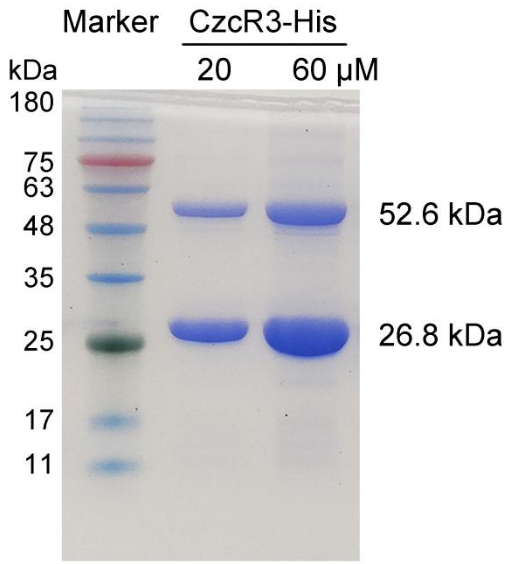

Figure S1. CzcR3 forms a homodimer *in vitro*. The purified His-tagged CzcR3 was diluted to 20 and 60 μM, and then mixed with an equal volume of loading buffer (250 mM Tris-HCl at pH 6.8, 2% SDS, 0.1% bromophenol blue, 20% glycerol, without dithiothreitol (DTT) or β-mercaptoethanol). 20 μl of the sample was electrophoresed in SDS-polyacrylamide gel (SDS-PAGE), and the gel was stained with coomassie brilliant blue. The molecular weight of CzcR3-His monomer is about 26.8 kDa, and that of CzcR3-His homodimer is about 53.6 kDa.

References

1. Bagdasarian, M.; Lurz, R.; Ruckert, B.; Franklin, F.C.H.; Bagdasarian, M.M.; Frey, J.; Timmis, K.N. Specific-purpose plasmid cloning vectors. II. Broad host range, high copy number, RSF1010-derived vectors, and a host-vector system for gene cloning in *Pseudomonas*. *Gene* **1981**, *16*, 237-247.
2. Gao, W.; Liu, Y.; Giometti, C.S.; Tollaksen, S.L.; Khare, T.; Wu, L.; Klingeman, D.M.; Fields, M.W.; Zhou, J. Knock-out of SO1377 gene, which encodes the member of a conserved hypothetical bacterial protein family COG2268, results in alteration of iron metabolism, increased spontaneous mutation and hydrogen peroxide sensitivity in *Shewanella oneidensis* MR-1. *BMC Genomics* **2006**, *7*, 76.
3. Nie, H.L.; Xiao, Y.J.; Liu, H.Z.; He, J.Z.; Chen, W.L.; Huang, Q.Y. FleN and FleQ play a synergistic role in regulating *lapA* and *bcs* operons in *Pseudomonas putida* KT2440. *Env. Microbiol. Rep.* **2017**, *9*, 571-580.
4. Liu, H.Z.; Xiao, Y.J.; Nie, H.L.; Huang, Q.Y.; Chen, W.L. Influence of (p)ppGpp on biofilm regulation in *Pseudomonas putida* KT2440. *Microbiol. Res.* **2017**, *204*, 1-8.
